# An optimised chromatin immunoprecipitation (ChIP) method for starchy leaves of *Nicotiana benthamiana* to study histone modifications of an allotetraploid plant

**DOI:** 10.1007/s11033-020-06013-1

**Published:** 2020-11-25

**Authors:** Buddhini Ranawaka, Milos Tanurdzic, Peter Waterhouse, Fatima Naim

**Affiliations:** 1 Centre for Agriculture and Bioeconomy, Institute for Future Environments, Queensland University of Technology, Brisbane, QLD 4000 Australia; 2 School of Biological Sciences, The University of Queensland, St Lucia, QLD 4072 Australia; 3 Centre for Crop and Disease Management, School of Molecular and Life Sciences, Curtin University, Bentley, WA 6102 Australia

**Keywords:** *Nicotiana benthamiana*, Histone modifications, ChIP-seq, Nuclei isolation, H3K4me3, H3K9me2

## Abstract

All flowering plants have evolved through multiple rounds of polyploidy throughout the evolutionary process. Intergenomic interactions between subgenomes in polyploid plants are predicted to induce chromatin modifications such as histone modifications to regulate expression of gene homoeologs. *Nicotiana benthamiana* is an ancient allotetraploid plant with ecotypes collected from climatically diverse regions of Australia. Studying the chromatin landscape of this unique collection will likely shed light on the importance of chromatin modifications in gene regulation in polyploids as well its implications in adaptation of plants in environmentally diverse conditions. Generally, chromatin immunoprecipitation and high throughput DNA sequencing (ChIP-seq) is used to study chromatin modifications. However, due to the starchy nature of mature *N. benthamiana* leaves, previously published protocols were unsuitable. The higher amounts of starch in leaves that co-precipitated with nuclei hindered downstream processing of DNA. Here we present an optimised ChIP protocol for *N. benthamiana* leaves to facilitate comparison of chromatin modifications in two closely related ecotypes. Several steps of ChIP were optimised including tissue harvesting, nuclei isolation, nuclei storage, DNA shearing and DNA recovery. Commonly available antibodies targeting histone 3 lysine 4 trimethylation (H3K4me3) and histone 3 lysine 9 dimethylation (H3K9me2) histone modifications were used and success of ChIP was confirmed by PCR and next generation sequencing. Collectively, our optimised method is the first comprehensive ChIP method for mature starchy leaves of *N. benthamiana* to enable studies of chromatin landscape at the genome-wide scale.

**Electronic supplementary material:**

The online version of this article (10.1007/s11033-020-06013-1) contains supplementary material, which is available to authorized users.

## Introduction

*Nicotiana benthamiana* is a plant species endemic to Australia first discovered by Benjamin Bynoe in 1839 [1]. It is an important biotechnological tool and a model plant for economically important crop family *Solanaceae*, which includes potato, tomato, peppers and eggplant [2]. The *N. benthamiana* ecotype used in laboratories all over the world is referred to as the ‘Lab’ isolate and another five wild isolates were collected from climaticaly diverse parts of Australia [2, 3]. Following the polyploidisation processes, *N. benthamiana* may have successfully passed the initial stage of genome instability and entered the prolonged phase of genome evolution referred to as diploidisation. In this phase, duplicated genes, chromosomes or chromosome fragments are progressively lost or retained due to the combined modifications in genetic and epigenetic structures [4–6]. Therefore, the species has an allotetraploid genome (~3.1 Gb) encoded in 19 chromosomes (aneutetraploid) [7]. It is considerably larger and more complex than the genome of the model plant *Arabidopsis thaliana* [8].

It is well established that histone modifications play a key role in regulation of biological processes [9] through their roles in the regulation of gene expression and genome integrity. Therefore, our understanding of the genetic control mechanisms involved in manipulation and expression of DNA is dependent on studies of the distribution of histone modifications in a genome [10]. Histone modifications that lead to active transcription are categorised as euchromatic marks while modifications responsible for gene suppression are categorised as heterochromatic marks [11]. In plants, some of these histone modifications mediate epigenetic regulation of gene expression underlying growth and development, including essential processes such as cell differentiation, floral transition, and gametogenesis [12].

The N-terminal tails of histones are exposed to a range of post-translational modifications such as methylation, phosphorylation, acetylation, ubiquitylation and sumoylation [13]. Some models have suggested that histone modifications can alter the interaction between histone-histone and histone-DNA through the action of protein complexes that recognise specific histone modifications and facilitate transcriptional activation or silencing [14, 15]. Allis and colleagues [16–18] proposed the ‘histone code’ hypothesis which refers to the sequential or combinatorial act of multiple histone modifications to regulate unique biological outcomes. This hypothesis describes three predictions, (1) different histone modifications could induce interactions with chromatin-associated proteins (2) histone modifications on the same or different histone tails might be interactive and produce diverse combinations on any one nucleosome and (3) the quality of higher order chromatin (euchromatin or heterochromatin) relies on the concentration and organisation of differentially modified nucleosomes. This ‘nucleosome code’ allows the assembly of different epigenetic states to allow diverse readouts of underlying genetic information such as gene activation or gene silencing [17]. Histone code hypothesis was proven to be correct with the identification of enzymes that recognise combinatorial patterns of histone marks [19]. The key proteins associated with these histone modifications are categorised as histone writers, readers and erasers. Histone writers are a group of enzymes capable of modifying specific amino acid residues on histone N-terminal tails where erasers remove these marks. Histone readers have specialised domains that can bind to specific histone marks and direct a particular transcriptional outcome [20, 21].

In plants, genome-wide analysis of histone post-translational modifications showed that histone 3 lysine 4 trimethylation (H3K4me3), histone 3 lysine 36 dimethylation (H3K36me2) and histone 3 lysine 36 trimethylation (H3K36me3) are enriched in highly expressed genes. These modifications were distributed throughout the gene with H3K4me3 enriched at the promoter and 5´ end of the genes [22] and H3K36me2 and H3K36me3 enriched in the transcribed regions [23, 24]. Another active mark is H3K4me2, a gene specific histone mark found at the 5ˊ end and promoter of active genes [25, 26]. However, the presence of H3K4me2 does not always correlate with active transcription [27–29]. Histone 3 lysine 9 acetylation (H3K9ac) is a euchromatic mark directly associated with active gene transcription and elongation [30, 31]. The heterochromatic mark, H3K9me2, is involved with transcriptional silencing of transposons and repetitive sequences, enriched over the promoter and gene body [32]. Another gene silencing mark is histone 3 lysine 27 trimethylation (H3K27me3) found in tissue specific and developmentally regulated genes and it is enriched along the gene body [31].

The genomic localisation of histone modifications in plant genomes can be determined through chromatin immunoprecipitation followed by high-throughput DNA sequencing (ChIP-seq). This method allows for genome-wide mapping of transcription factors, DNA binding proteins and histone modifications. ChIP protocols were initially developed for yeast [33], *Drosophila* [34] and then for mammalian cells [35]. These reported protocols are not always directly applicable to plant tissues due to structural and biochemical differences between plant and animal cells [36]. Plant cells have rigid cell walls, larger vacuoles, and higher levels of lignin and cellulose. Therefore, ChIP protocols have been heavily modified to extract high quality chromatin from plant tissues [37]. Generally, in a ChIP-seq workflow, formaldehyde is used to crosslink DNA binding proteins to DNA (in the case of native ChIP, where no crosslinking is performed, this step can be omitted), followed by the fragmentation of chromatin to 200–1000 bp fragments, immunoprecipitation of soluble chromatin fragments with antibodies against a histone modification of interest, reverse crosslinking the immunoprecipitated complexes to release the DNA fragments and their preparation for next generation sequencing (NGS) [10, 36, 38].

*N. benthamiana* plants are used for rapid transient gene expression and metabolic engineering by agroinfiltration as well as to study plant–pathogen interactions. The collection of wild *N. benthamiana* isolates provides a unique resource to study histone modifications with implications on manipulation of gene homoeologs, response to environmental stresses and synthetic biology. Therefore, in this study we aimed to develop a reliable ChIP protocol for mature *N. benthamiana* leaves to determine the presence and distribution of gene regulatory histone modifications. The majority of published plant ChIP protocols are developed for seedlings and young plant tissues [12, 36, 37, 39–41] and we found that the methods developed for Arabidopsis [39] and tomato leaves [41] were unsuitable for ChIP of mature *N. benthamiana* leaves. Here, we use a combination of nuclei isolation and chromatin immunoprecipitation protocols to enable studies of chromatin landscape of mature *N. benthamiana* leaves.

## Methods

### Plant material

*Nicotiana benthamiana* plants of Lab and Qld ecotypes were grown on soil (Plugger custom Mix, supplemented with Osmocote^®^ slow release fertiliser) under controlled environmental conditions of 25 ºC and a 16 h photoperiod. *N. benthamiana* Lab is the commonly used ecotype and Qld refers to the ecotype collected from Queensland in Australia [3]. Leaves of 5 weeks old plants were collected for crosslinking at the beginning of their photoperiod.

### Crosslinking of DNA and protein

Two leaves (~3 g) of 5 weeks of old *N. benthamiana* plants (~5.5 × 5.0 cm) were rinsed twice with 40 mL of water and patted dry using paper towel, transferred to Falcon tube containing 37 mL of 1% pre-chilled formaldehyde (Sigma-Aldrich, 252,549), vacuum infiltrated for 10 min at −25 in Hg. The amount of tissue should not exceed one Falcon tube. Crosslinking was stopped by addition of 2.5 mL of 2 M glycine to a final concentration of 0.125 M, solutions were mixed well, and vacuum infiltration continued for 5 min at −25 in Hg. It is critical that the leaf tissues remain in solution throughout the vacuum infiltration process. The buffers were removed, crosslinked materials were rinsed with milliQ water and any excess water was removed thoroughly using paper towels.

### Chromatin isolation from crosslinked tissue

All buffers were prepared except for the following steps carried out just before nuclei isolation. Sodium metabisulfite was added to NEB (nuclei extraction buffer containing 0.5 M Mannitol, 10 mM PIPES-KOH, 10 mM MgCl_2_, 2% PVP40, 200 mM l-lysine monohydrochloride, 6 mM EGTA) to a final concentration of 10 mM. NEB complete buffer was prepared by the addition of β-mercaptoethanol to half of NEB to a final concentration of 0.4 mM. 150 mL of ice-cold NEB complete buffer was poured into a Waring blender containing 4–5 g of crosslinked leaf tissue and homogenised for 30 s in low setting. The homogenate was filtered through 4 layers of cheesecloth into a 250 mL sterile glass beaker on ice followed by second filtration through 4 layers of miracloth into a 250 mL sterile glass measuring cylinder. The volume was adjusted to 147 mL with NEB complete buffer followed by addition of 3 mL of 25% Triton X-100 (prepared with NEB complete buffer), cylinder sealed with parafilm and mixed very gently by inversion 10–20 times. Homogenate was aliquoted into three 50 mL Falcon tubes and spun down at 57 g at 4 ºC for 2 min. The pellet was discarded, and supernatant was transferred to a new set of tubes and spun down at 1800 g for 15 min at 4 ºC. After centrifugation supernatant was discarded and each pellet was resuspended in 50 mL NEB. Contents were mixed gently by inversion until the pellet was completely resuspended and spun down again at 1800 g for 15 min at 4 ºC. The supernatant was discarded, pellet resuspended in 5 mL of NEB and all resuspended nuclei pellets were combined into one Falcon tube to a final volume of 50 mL with NEB. Tubes were spun down again as before, supernatant was discarded and the nuclei fraction was resuspended in 1.5 mL of nuclei storage buffer (20% Glycerol, 20 mM HEPES–KOH (pH 7.2), 5 mM MgCl_2_ and 1 mM DTT). The nuclei can either be stored at −80 ºC for later use or proceed with nuclei lysis and DNA shearing.

### Nuclei lysis and chromatin shearing

An aliquot of nuclei resuspension (750 uL) was transferred into microcentrifuge tubes and centrifuged at 1000 rpm for 10 min. The supernatant was discarded, and the pellet was resuspended in 300 uL of freshly prepared nuclei lysis buffer (50 mM Tris–HCl, pH 8, 10 mM EDTA, 1% SDS, 50 mM Protease Inhibitor (Roche cOmplete Tablets, Mini EDTA-free, EASYpack 04693159001). A 5 uL aliquot from each sample was set aside for verifying the efficiency of chromatin shearing.

To compare shearing techniques, 100 uL of sample was sheared with Diagenode Bioruptor^®^ and 130 uL of sample transferred into microtubes (520045-microTUBE Snap-Cap, AFA Fibre) for shearing with Covaris M220^™^ Focused-ultrasonicator with SonoLab^™^7.2. The power was set to “High” in Diagenode Bioruptor^®^ and a time course of shearing cycles (14, 16, 18, 20 and 22 cycles) (30 s “ON”, 30 s “OFF”) was conducted. Chromatin shearing using Covaris sonicator was carried out using the “150 bp DNA 130 uL microTUBE” program selected with Min Temp of—18 ºC, Set Point temperature of—20 ºC and Max Temp—22 ºC, treatment was at Peak power—50.0, Duty factor—20.0 and Cycles/Burst—200. Shearing was completed in 5 min and 32 s per sample.

The sonication efficiency was assessed using 5 uL of sheared chromatin with 5 uL intact DNA. DNA samples were treated with RNase A (1 uL of 10 mg/mL RNase A, ThermoFisher Scientific, EN0531), 2 uL of NEB restriction enzyme buffer 2 (NEBuffer 2, B7002S), 8 uL of deionised water and incubated at 37 ºC for 30 min followed by the addition of 2 uL of 20 mg/mL Proteinase K (Promega V3021), 1 uL of 1M Tris–HCL (pH 6.5), and incubation at 45 ºC for 15 min. Samples were electrophoresed on 1.2% agarose gel in 1× TAE buffer at 100 V for 45 min (Fig. 2a, b). The samples used for ChIP were sheared using Covaris sonicator only. (Fig. 2b).

### Chromatin immunoprecipitation

Sonicated chromatin was centrifuged at 12,000 rpm at 4 ºC for 5 min and the supernatant was transferred to a new tube. An aliquot of each sample (20 uL) was set aside to serve as the ‘input’ DNA control. Volumes of chromatin samples were measured and adjusted to 1.5 mL by adding freshly prepared ChIP dilution buffer [1.1% Triton X-100, 1.2 mM EDTA, 16.7 mM Tris–HCl (pH 8), 167 mM NaCl]. Chromatin solutions were split into three tubes (500 uL each) that corresponded to the number of antibodies tested [Commercially available rabbit polyclonal antibodies against histone H3K4me3 (Abcam ab8580) and H3K9me2 (Diagenode C15410060)] and the ‘no antibody control’ (NAB). For six treatments (including two replicates per antibody, input and NAB samples) 60 uL of magnetic beads (Dynabeads^™^ Protein A 10002D) were washed twice with 100 uL of ChIP dilution buffer, resuspended in 60 uL of ChIP dilution buffer and 10 uL was transferred to each chromatin sample referred to as immunoprecipitation (IP) sample. Tubes were rotated in a tube rotator at 4 ºC for 1 h. Meanwhile, 120 uL aliquot of magnetic beads was washed twice with 150 uL of phosphate buffer (0.1 M Na_2_HPO_4,_ 5 mM NaH_2_PO_4_, pH 8.1), resuspended in 120 uL of the same buffer and 20 uL added to each IP sample. A 5 uL aliquot of the appropriate antibody was added to each IP samples (water for the NAB samples) and tubes were rotated in a tube rotator at 4 ºC for 1 h. Beads were captured in precleared chromatin and the supernatant was transferred into corresponding tubes containing antibody-bead complex. Contents were mixed by pipetting and rotated overnight at 4 ºC. Beads were recovered using a magnetic stand and the supernatant was removed. Beads were washed three times sequentially (Table S1 in Online Resource 1) with Low salt wash buffer [150 mM NaCl, 0.1% SDS, 1% Triton X-100, 2 mM EDTA, 20 mM Tris–HCl (pH 8)], High salt wash buffer [500 mM NaCl, 0.1% SDS, 1% Triton X-100, 2 mM EDTA, 20 mM Tris–HCl (pH 8)], LiCl wash buffer [0.25 mM LiCl, 1% IGEPAL, 1% Sodium deoxycholate, 1 mM Tris–HCl (pH 8)] and TE buffer [100 mM Tris–HCl (pH 8), 10 mM EDTA].

Immunoprecipitated complex was eluted in 175 uL of TES buffer [100 mM Tris–HCl (pH 8), 10 mM EDTA, 1% SDS] followed by brief vortexing and incubation at 65 ºC for 15 min with gentle agitation. The beads were captured using magnetic stand and supernatant was carefully transferred to a new tube. This step was repeated with a second elution in 175 uL of TES and the two elutes of each sample were combined. At this stage 350 uL of TES was added to ‘input’ DNA samples.

### Reverse crosslinking, DNA recovery and ChIP validation

To reverse crosslink, 20 uL of 5 M NaCl was added to each sample including ‘input’ DNA control samples and incubated overnight at 65 ºC. All samples were treated with 2 uL of 10 mg/mL RNase A (ThermoFisher Scientific EN0531), and incubated at 37 ºC for 30 min followed by the addition of 1 uL of 20 mg/mL Proteinase K (Promega V3021), 10 uL of 0.5 M EDTA, 20 uL of 1 M Tris–HCl (pH 6.5) and incubation at 45 ºC for 1 h. Samples were cleaned using phenol:chloroform (1:1, v/v). One volume of phenol:chloroform (1:1, v/v) was added to each sample, mixed thoroughly and centrifuged at room temperature for 5 min at 14,000 rpm. The upper aqueous layer was transferred to a clean tube followed by addition of 0.1× total sample volume of 3 M NaOAc and 2.5× supernatant volume of 100% ethanol and DNA precipitated overnight at −20 ºC. Samples were centrifuged for 30 min at 14,000 rpm at 4 ºC, supernatant discarded and 500 uL of 70% ethanol added to each tube followed by centrifugation at 14,000 rpm for 5 min at 4 ºC. Supernatant was carefully removed, DNA pellet air dried and resuspended in 20 uL of nuclease free water. A second column clean-up was carried out using MinElute^®^ Reaction Cleanup kit (QIAGEN 28204) as per manufacturer’s instructions and DNA eluted in 15 uL of elution buffer.

For ChIP DNA validation, 1 uL of input DNA and ChIP DNA were diluted in 50 uL and 10 uL of TE buffer, respectively. Primer pairs were designed using the latest assembly of *N. benthamiana* genome (http://www.nbenth.com) to specifically amplify a ~200 bp fragment of *Ef-1a* (active gene to probe success of ChIP with H3K4me3 [42], gene ID Nbv6.1trA73553 and *Ty1-copia* (retrotransposon to probe success of ChIP with H3K9me2 [43], gene ID Nbv6.1trA2043) (Table S2 in Online Resource 2). PCR was set up using 1.5 uL of the diluted extract as template in 2X 2G Robust HotStart ReadyMix (KAPA Biosystems KK5704). The PCR cycle conditions were as follows: an initial denaturation step at 95 °C for 3 min, 30 cycles of 95 °C for 15 s, 60 °C for 15 s, and 72 °C for 30 s, and a final extension step at 72 °C for 2 min. The resulting PCR products were electrophoresed on a 1% TAE agarose gel (Fig. 3a).

### ChIP-seq library preparation

ChIP DNA concentrations were determined using Qubit^®^2.0 Fluorometer. Libraries were prepared with 2 ng of ChIP DNA using NEBNext^®^ Ultra^™^ II DNA Library Prep Kit for Illumina (E7645S) as per manufacturer’s specifications. Libraries were quantified using Qubit^®^2.0 Fluorometer and qPCR methods as per Library Quantification Kit Illumina^®^ Platforms (KAPA Biosystems) (Table S3 in Online Resource 3). The final concentration of libraries ranged between 17 and 63 ng/uL and an aliquot of each library was electrophoresed on a 1.5% agarose gel. The size of the fragments ranged between 200 and 500 bp (Fig. 3b), which was further confirmed by LabChip GX (Caliper Life Sciences) (Fig. 3c). Libraries were sequenced at the Central Analytical Research Facility (CARF), Queensland University of Technology, using Illumina NextSeq^®^ 500 with output of 75 bp paired-end reads (TG NextSeq^®^ 500/550 High Output Kit v2, 75 cycle, TG-160-2005).

## Results

### Formaldehyde vacuum infiltration

**Fig. 1:**
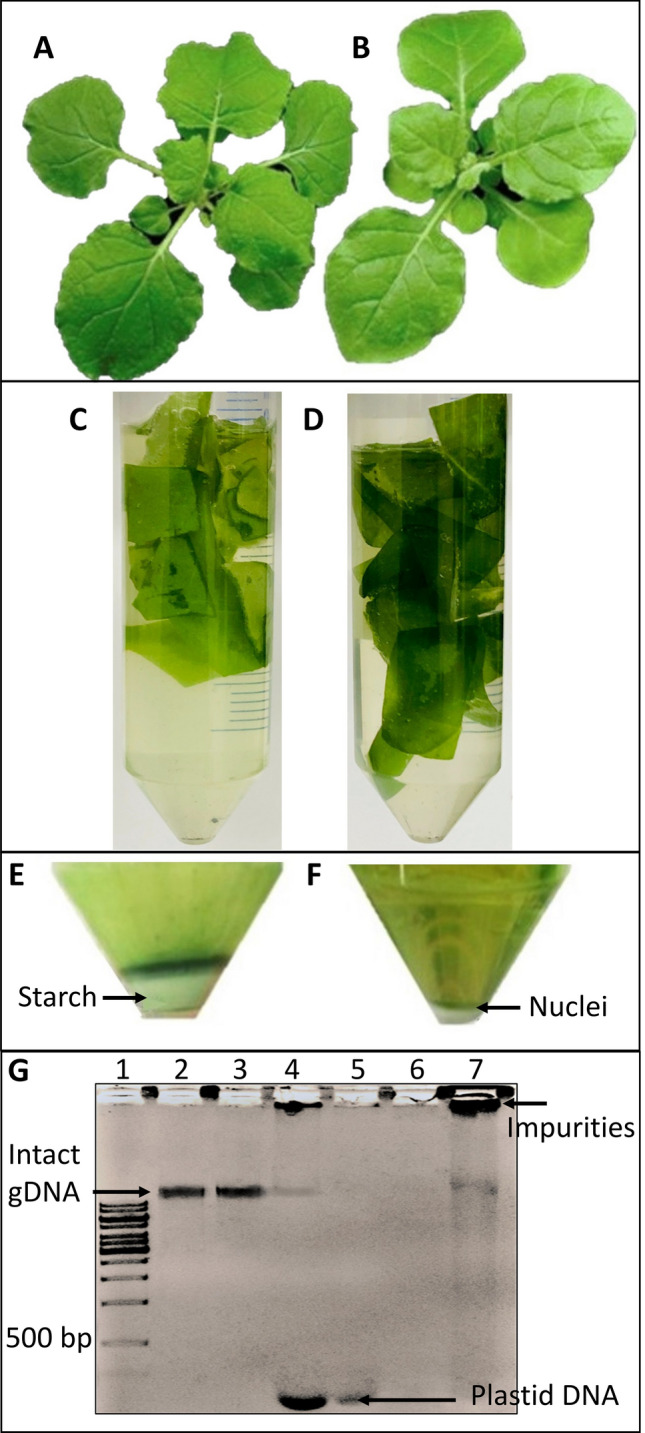
*N. benthamiana* leaf samples used for crosslinking and nuclei extraction. A representative five weeks old *N. benthamiana* plant of Lab (**a**) and Qld (**b**) ecotypes used for ChIP. Excised leaf samples were sub-merged in formaldehyde for crosslinking (**c**) and after vacuum infiltration appeared translucent, water-soaked and sunken at the bottom of the tube (**d**). Chromatin extraction using previously published protocols results in large amounts of starch co-precipitating with DNA (**e**) and significant reduction in starch with nuclei preparation optimised for mature *N. benthamiana* leaves (**f**). Comparison of quality and quantity of chromatin extracted with modified and conventional methods (**g**), Lane 1: GeneRuler^™^ 1 kb DNA Ladder, Lanes 2 and 3 contain Lab and Qld genomic DNA (gDNA) extracted using modified method. Lanes 4 and 5 contain Lab gDNA and lanes 6 and 7 contain Qld gDNA extracted using conventional method

Freshly prepared 1% formaldehyde is used for crosslinking and this was found to be appropriate for mature leaves of *N. benthamiana* (Fig. 1a, b). The success of formaldehyde vacuum infiltration was determined by the obvious physical changes in leaf samples. Before crosslinking, leaf samples floated on the surface of 1% formaldehyde solution, and the abaxial and adaxial surfaces of the leaf tissues differed in colour (Fig. 1c). After crosslinking, tissues sunk to the bottom of the tube and looked translucent and water-soaked (Fig. 1d) indicating the success of crosslinking.

### Removal of starch contamination in nuclei extract

The next step of a conventional ChIP-seq workflow requires chromatin extraction from the crosslinked material. With conventional methods [39, 44] we noticed that a large amount of starch co-precipitated with chromatin (Fig. 1e). This reduced the final quality and quantity of precipitated DNA and samples were contaminated with starch, proteins and plastid DNA (Fig. 1e, g). In comparison, the modified protocol described here yielded high quality nuclei (Fig. 1f) which resulted in high quality chromatin preparation (Fig. 1g and Table S4 in Online Resource 4). The described method is a combination of *N. benthamiana* nuclei isolation methods [44, 45] and ChIP methods developed for *Arabidopsis* and tomato [36, 39, 41]. To further eliminate starch contamination, leaves were harvested after the dark period.

### Successful shearing of chromatin

**Fig. 2:**
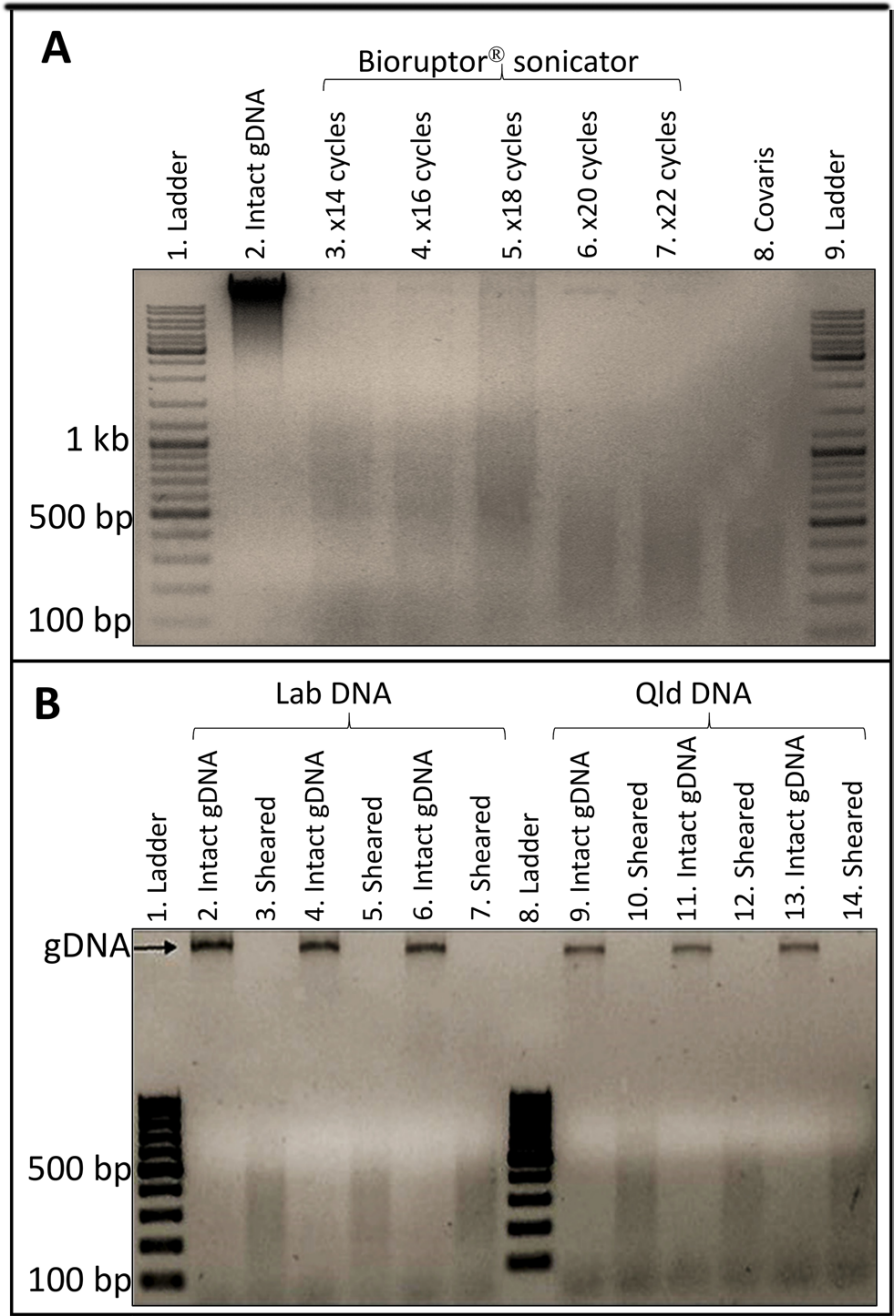
Comparison of DNA shearing efficiency using Bioruptor sonicator and Covaris ultrasonicator. **a** Optimisation of the number of sonication cycles required with Bioruptor^®^ sonicator to fragment gDNA compared to Covaris ultrasonicator. The desired fragmentation range (100–800 bp) achieved with 20 or 22 cycles using Biorupter whereas with Covaris the 150 bp instrument setting was sufficient. **b** Three biological replicates of Lab and Qld intact gDNA sheared with Covaris ultrasonicator using the 150 bp instrument setting to generate reproducible fragment range in all replicates

The next crucial step in ChIP protocol is chromatin shearing and generally a Bioruptor^®^ sonicator is used. We tested different number of sonication cycles (14, 16, 18, 20, 22) to determine the optimum number of cycles to achieve the required sheared DNA fragment range (200–500 bp). This was achieved with 20 and 22 cycles and the lower number of sonication cycles resulted in incomplete shearing and larger fragment sizes (Fig. 2a). We then compared this to shearing efficiency of Covaris M220 ultrasonicator which produced the desired chromatin shearing results with narrow fragment range and no requirement for optimisation (Fig. 2a, b). The efficiency of shearing was confirmed by electrophoresing an aliquot of each sample on an agarose gel. With a clear difference obvious between the sheared and input samples using both techniques (Fig. 2a). The fragmentation was consistent between the biological replicates used for Lab and Qld samples (Fig. 2b).

### Chromatin immunoprecipitation and validation

**Fig. 3:**
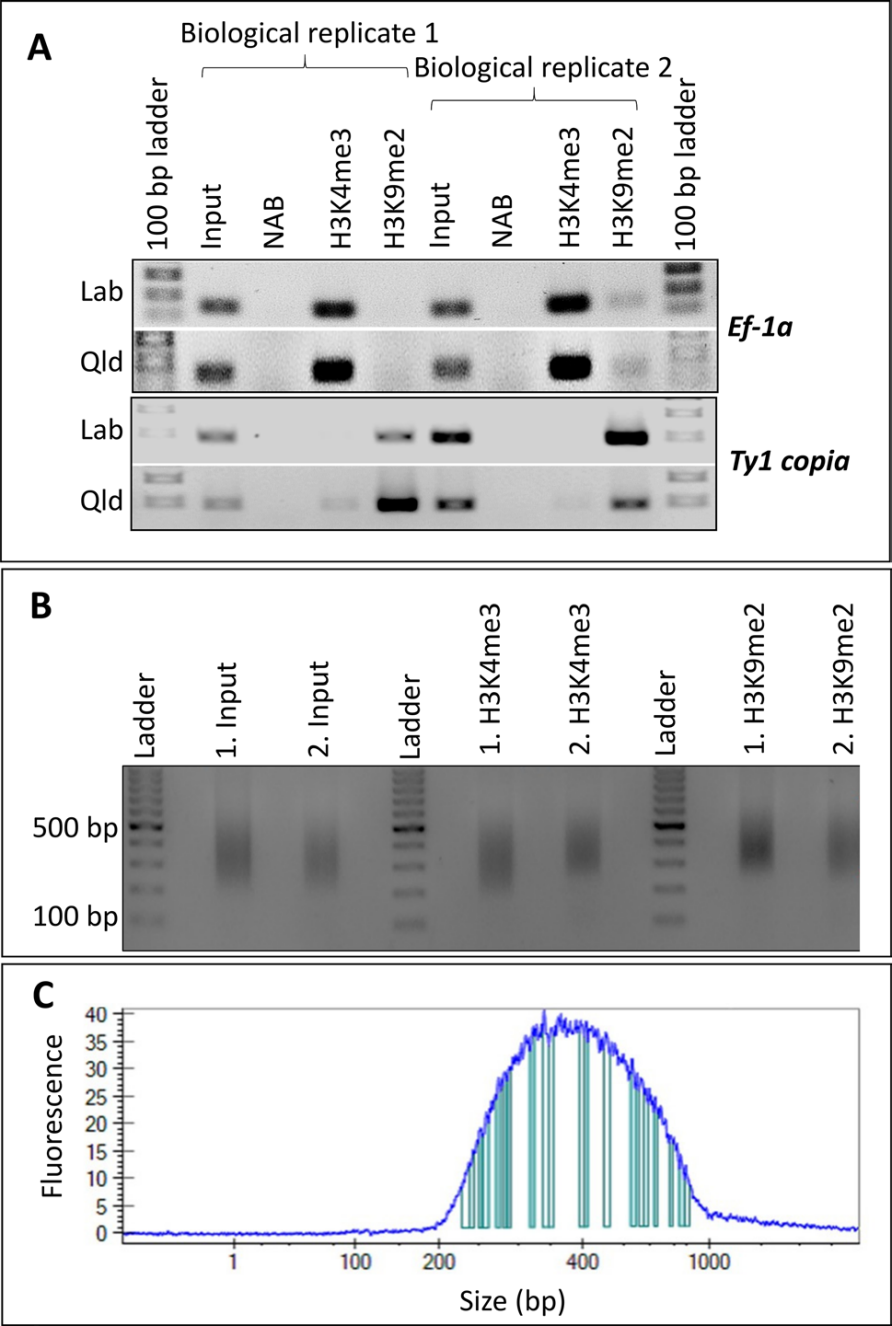
PCR analysis of regions enriched with H3K4me3 and H3K9me2 and quality checks of ChIP libraries prepared for next generation sequencing. **a** Primer pairs used for amplification of ~200 bp region of EF-1a and Ty1-copia in H3K4me3 and H3K9me2 ChIPs, respectively. Input: DNA extracted from the nuclei before the immunoprecipitation step. *NAB* no antibody control. H3K4me3 and H3K9me2: ChIP DNA. 1.5 μL of 1/50 diluted input and 1/10 diluted ChIP samples used for PCR. **b** An aliquot of each ChIP library electrophoresed on 1.5% agarose gel. **c** A representative Bioanalyzer chromatogram of a ChIP library to confirm fragment size of the ChIP library

The success of chromatin immunoprecipitation is confirmed by PCR which were carried out for *N. benthamiana* IP samples using primer pairs specific to elongation factor 1 alpha (*EF-1a*) and *Ty1-copia* retrotransposon (Fig. 3a). The histone modification statuses of *EF-1a* gene and *Ty1-copia* retrotransposon are expected to be enriched in H3K4me3 and H3K9me2 marks, respectively [42, 43]. The IPs were validated for active and inactive genes. A slight non-specific amplification was also observed, and this may have been due to the allotetraploid genome of *N. benthamiana* with large gene families and partially resolved gene activity of homoeologs. Most genes have two homoeologs and in some instances one copy is active and the other copy is aberrant or inactive [2]. For such gene homoeologs and specially for *EF-1a*, a gene with many copies, it is possible to see weak amplification in H3K9me2. Willing and colleagues [46] have shown that copia elements are enriched in H3K4me3 ChIP which explains the slight amplification of *Ty1-copia* in H3K4me3 ChIP.

### ChIP-seq library preparation and data analysis

The concentration of DNA in IP samples was quantified using Qubit^®^ prior to library preparation (Table S5 in Online Resource 5). As expected, the input control samples had a higher concentration compared to the IP samples. The concentration of DNA in IP samples enriched for H3K4me3 and H3K9me2 marks were between 2 and 4 ng/uL which was sufficient for library preparation. Libraries were successfully prepared and Illumina NextSeq^®^ 500 platform was used for sequencing.

**Fig. 4:**
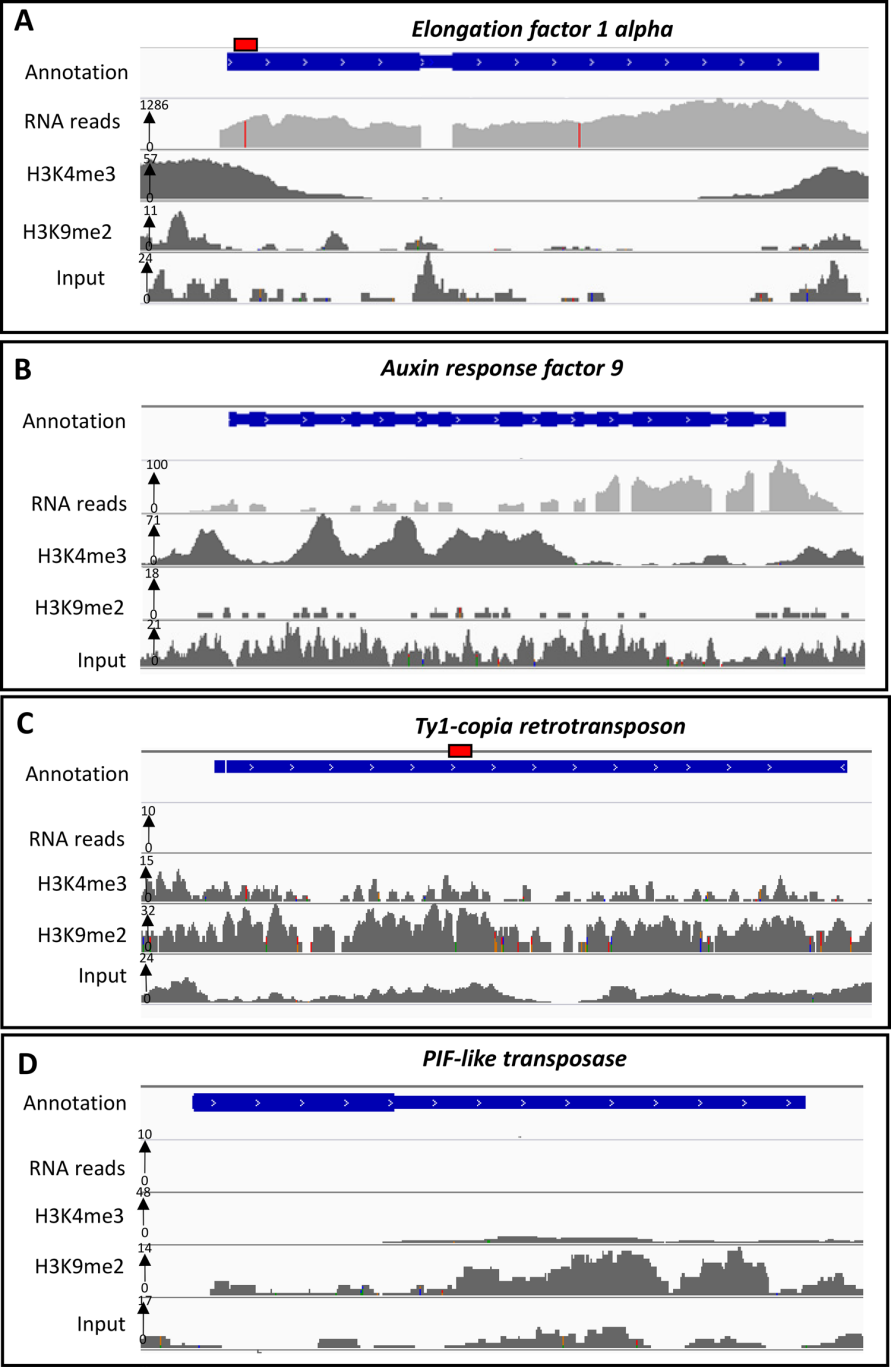
Integrative Genomics Viewer (IGV) used to visualise H3K4me3 and H3K9me2 tracks aligned to *N. benthamiana* genome scaffolds for representative active and repressed genes (*Ef-1a*, *Auxin response factor 9 Ty1-copia* and *PIF-like transposase*). The H3K4me3 and H3K9me2 files used for visualisation were generated using bamCompare tools. The tool normalises and compares two BAM files (Input and ChIP) to obtain the log2ratio or difference between them. An overlay of RNA reads obtained from equivalent aged *N. benthamiana* leaves is also provided. **a**, **b** ChIP-seq tracks displaying the fold enrichment and distribution of H3K4me3 over transcriptionally active genes, *Ef-1a* and *Auxin response factor 9*, in *N. benthamiana* genome. **c**, **d** ChIP-seq tracks displaying the fold enrichment and distribution of H3K9me2 over a transcriptionally repressed genes, *Ty1-copia* and *PIF-like transposase*, in *N. benthamiana* genome. The red boxes on *Ef-1a* and *Ty1-copia* gene annotations are indicative of the PCR amplified regions for ChIP validation shown in Fig. 3

Quality control checks of raw reads were carried out using FastQC (Table 1) [47] and poor-quality reads were removed using Trimmomatic [48]. After read alignment using Bowtie2 [49] to latest assembly of *N. benthamiana* genome (http://www.nbenth.com), DeepTools [50] was used to normalise IP reads against control input reads and the results (files generated by bamCompare tool) were visualised on Integrative Genomics Viewer (IGV) [51] (Fig. 4). The differential distribution of H3K4me3 and H3K9me2 can be clearly observed over two selected transcriptionally active genes (*Ef-1a* and *Auxin response factor 9*) (Fig. 4a–b) and two repressed regions (*Ty1-copia* and *PIF-like transposase*) (Fig. 4c, d) in the *N. benthamiana* genome, respectively. H3K4me3 is highly enriched over the transcriptionally active genes while H3K9me2 is enriched over transcriptionally repressed genes, and transposable elements and repeats, proving the success and accuracy of the modified method. RNA sequencing reads (available from Waterhouse laboratory) from mature leaves of *N. benthamiana* are also overlayed with the ChIP peaks to further confirm ChIP-seq data. We estimated the reproducibility between ChIP-seq replicate pairs by calculating the Spearman correlation coefficients [50]. The correlation between replicate pairs were high (R > 0.9), indicating the consistency and reproducibility (Table S6 in Online Resource 6).

**Table 1:** Basic sequencing quality control parameters of ChIP-seq libraries

Ecotype	Sample	Antibody	Sequences flagged as poor quality	% GC	% Over-represented sequences	Adapter content	Raw reads (millions)	Mapped reads (millions)	Uniquely mapped reads (millions)
Lab	Rep 1	Input	0	37	0.11	0	178	135	43
	Rep 2	Input	0	37	0.00	0	124	120	54
	Rep 1	H3K4me3	0	43	0.10	0	101	73	48
	Rep 2	H3K4me3	0	40	0.10	0	103	78	44
	Rep 1	H3K9me2	0	40	0.20	0	320	288	151
	Rep 2	H3K9me2	0	40	0.10	0	310	282	122
Qld	Rep 1	Input	0	38	0.11	0	112	109	47
	Rep 2	Input	0	38	0.20	0	128	125	53
	Rep 1	H3K4me3	0	42	0.11	0	98	75	47
	Rep 2	H3K4me3	0	43	0.11	0	87	72	44
	Rep 1	H3K9me2	0	41	0.11	0	270	264	149
	Rep 2	H3K9me2	0	39	0.00	0	235	220	108

## Discussion

To enable studies of histone modifications in polyploid, *N. benthamiana*, we combined nuclei isolation [44, 45] and ChIP methods [36, 39, 41]. Various steps including tissue harvesting, nuclei isolation, nuclei storage, chromatin shearing and ChIP DNA recovery were optimised to yield high quality ChIP DNA suitable for next generation sequencing. Generally, 5–6 weeks old *N. benthamiana* Lab isolate plants are used for genetic manipulation to study plant–microbe interactions, metabolic pathways, vaccine production and synthetic biology [2, 3]. A comparative analysis of *N. benthamiana* ecotypes collected from climatically harsh and diverse locations of Australia are also suitable for studying biotic and abiotic stress due to their adaptation [2]. Therefore, we sought to develop a ChIP-seq protocol for mature leaves of 5 weeks old *N. benthamiana* plants.

The first critical step in plant ChIP method is crosslinking. The leaf tissues were fixated in formaldehyde due to its cell permeability, rapid reactivity and reversibility over other crosslinking agents [52]. Freshly diluted formaldehyde solution was used for crosslinking [53] to avoid oxidation products of formaldehyde such as formic acid and paraformaldehyde. Furthermore, to enable successful reversal of crosslinking, it is crucial to use 1% formaldehyde and to not exceed the duration of vacuum infiltration. An appropriate amount of leaf tissue was used for complete submergence in the formaldehyde solution throughout the duration of vacuum infiltration [37]. The process of vacuum infiltration replaces the air inside mesophyll cells of the leaf tissues with aqueous formaldehyde [41]. Therefore, completion of fixation is assessed by the translucent and water-soaked appearance of tissue.

Traditionally, plant ChIP protocols are laborious and time consuming due to buffer preparation, chromatin isolation and DNA shearing [41, 54]. In this method, only a single nuclei extraction buffer (NEB) was necessary for successful isolation of nuclei. To enable storage of nuclei prep for future use and suitable for other sequencing techniques, an extra step was added by resuspending nuclei in nuclei storage buffer and storage at -80 ºC. DNA fragmentation was also optimised by using the versatile Covaris ultrasonicator which takes 5 min and 32 s to shear DNA in contrast to previous report of *N. benthamiana* leaf ChIP requiring 60 min sonication with Bioruptor^®^ sonicator [55]. The sonication step with Bioruptor^®^ sonicator also initially requires fragmentation monitoring by gel electrophoresis to empirically identify the optimum number of sonication cycles [38, 56]. Although other sonicators may serve a similar purpose to achieve the intended fragmentation outcome, by using Covaris ultrasonicator we were able to generate reproducible fragmentation for all of *N. benthamiana* samples with no optimisation required.

Another critical step to enable high quality ChIP-seq library preparation is to eliminate buffer salts and phenol. Traditionally, DNA is extracted once with phenol/chloroform (1:1, v/v) and recovered by precipitation with ethanol after reversal of crosslinking. This technique is appropriate for PCR based analysis however not ideal for next generation sequencing. Our optimised protocol uses phenol/chloroform (1:1, v/v) followed by a second clean up with QIAGEN affinity column. Although a second clean up may not be necessary, based on the low recovery of ChIP and potential interference of carbohydrates, polysaccharides, and phenol in inhibiting library amplification [57] it would be ideal to use a column clean product prior to library preparation. Silica membrane spin columns are recommended to remove carbohydrates and polysaccharides. Although the modified method successfully reduces starch contamination, we opted for a second clean up using column to eliminate the risk of carryover carbohydrates and phenol [58]. The affinity column is suitable for clean-up of DNA fragments ranging between 70 bp and 4 kb.

We further confirmed the success of the protocol by analysing the H3K4me3 and H3K9me2 ChIP-seq results (Fig. 4). An overlay of histone modification peaks and RNA sequencing reads using representative genes from *N. benthamiana* genome confirmed the success of the protocol. Furthermore, the global genomic comparison of the sequenced libraries for H3K4me3 and H3K9me2 for the two *N. benthamiana* ecotypes are being analysed to understand the implications of chromatin landscape on the phenotypic and environmental adaptation of the species.

## Conclusion

Overall, we were able to prepare high quality ChIP libraries using mature leaves of *N. benthamiana*. The protocol described here allows for extraction of high-quality chromatin from mature leaves of *N. benthamiana* by reducing starch contamination. This will enable epigenetic research using the increasingly popular model plant, *N. benthamiana*, and comparisons to its wild ecotypes.

## Electronic supplementary material

Below is the link to the electronic supplementary material.

Supplementary file1 (DOCX 23 KB)

## Data Availability

The datasets analysed during the current study are available from the corresponding author on request.
