# Supplementary material for: An optimised chromatin immunoprecipitation (ChIP) method for starchy leaves of Nicotiana benthamiana to study histone modifications of an allotetraploid plant
Source: Mol Biol Rep. 2020 Nov 25;47(12):9499–509. doi: 10.1007/s11033-020-06013-1 (PMC7723940; doi:10.1007/s11033-020-06013-1)
Supplement: Supplementary file 1 — Supplementary file1 (DOCX 23 KB) [file 11033_2020_6013_MOESM1_ESM.docx]

**Table S1 Washing procedure of recovered magnetic beads**

| First Wash | Second Wash | Third Wash |
| --- | --- | --- |
| 1. Resuspend beads in 800 uL buffer 2. Remove supernatant using a magnetic stand | 1. Resuspend beads in 800 uL buffer 2. Wash beads for 5 minutes with rotation 3. Remove the supernatant using a magnetic stand | 1. Repeat second wash |

**Table S2 Primer sequences used to validate ChIP DNA**

| Locus | Forward primer sequence | Reverse primer sequence |
| --- | --- | --- |
| *EF-1a* | CCACGTCGACTCTGGTAAGT | AGCACCCAGGCATACTTGAA |
| *Ty1-copia* | AGGACATGTGAAGAAGCCGA | TTGTTAGCCACCATGTTCGC |

**Table S3 Library concentrations for IP and Input DNA samples**

| Ecotype | Sample | Antibodies | Library Concentration (ng/μL) |
| --- | --- | --- | --- |
| Lab | Rep 1 | Input | 37.30 |
|  | Rep 2 | Input | 33.00 |
|  | Rep 1 | H3K4me3 | 63.60 |
|  | Rep 2 | H3K4me3 | 17.10 |
|  | Rep 1 | H3K9me2 | 22.24 |
|  | Rep 2 | H3K9me2 | 31.60 |
| Qld | Rep 1 | Input | 21.20 |
|  | Rep 2 | Input | 40.20 |
|  | Rep 1 | H3K4me3 | 48.40 |
|  | Rep 2 | H3K4me3 | 38.40 |
|  | Rep 1 | H3K9me2 | 20.80 |
|  | Rep 2 | H3K9me2 | 17.40 |

**Table S4 Nanodrop determined 260/280 and 260/230 ratios for DNA samples (Fig. 1G) extracted using the modified and conventional methods**

| Extraction Method | Sample | 260/280 | 260/230 | Corresponding gel lanes shown in Fig. 1G |
| --- | --- | --- | --- | --- |
| Modified | Lab | 1.91 | 2.10 | 2 |
|  | Qld | 2.07 | 2.22 | 3 |
| Conventional | Lab 1 | 1.56 | 0.44 | 4 |
|  | Lab 2 | 0.97 | 0.30 | 5 |
|  | Qld 1 | 0.18 | 0.05 | 6 |
|  | Qld 2 | 1.51 | 0.09 | 7 |

**Table S5 Concentrations of *N.benthamiana* ChIP DNA recovered after antibody conjugation**

| Ecotype | Sample | Antibodies | Concentration of ChIP DNA (ng/μL) |
| --- | --- | --- | --- |
| Lab | Rep 1 | Input | 14.22 |
|  | Rep 2 | Input | 14.46 |
|  | Rep 1 | H3K4me3 | 2.56 |
|  | Rep 2 | H3K4me3 | 2.96 |
|  | Rep 1 | H3K9me2 | 3.24 |
|  | Rep 2 | H3K9me2 | 3.92 |
| Qld | Rep 1 | Input | 14.30 |
|  | Rep 2 | Input | 15.24 |
|  | Rep 1 | H3K4me3 | 2.06 |
|  | Rep 2 | H3K4me3 | 2.74 |
|  | Rep 1 | H3K9me2 | 3.08 |
|  | Rep 2 | H3K9me2 | 3.10 |

**Table S6 Correlation coefficient matrix of ChIP-seq samples**

| Ecotype | Sample | InputR1 | InputR2 | H3K4me3R1 | H3K4me3R2 | H3K9me2R1 | H3K9me2R2 |
| --- | --- | --- | --- | --- | --- | --- | --- |
| Lab | InputR1 | 1.00 | 0.92 | 0.78 | 0.81 | 0.67 | 0.69 |
|  | InputR2 | 0.92 | 1.00 | 0.83 | 0.89 | 0.77 | 0.80 |
|  | H3K4me3R1 | 0.78 | 0.83 | 1.00 | 0.93 | 0.63 | 0.68 |
|  | H3K4me3R2 | 0.80 | 0.89 | 0.93 | 1.00 | 0.71 | 0.76 |
|  | H3K9me2R1 | 0.66 | 0.77 | 0.63 | 0.71 | 1.00 | 0.96 |
|  | H3K9me2R2 | 0.69 | 0.80 | 0.68 | 0.77 | 0.96 | 1.00 |
| Qld | Input R1 | 1.00 | 0.91 | 0.60 | 0.64 | 0.66 | 0.67 |
|  | InputR2 | 0.91 | 1.00 | 0.69 | 0.72 | 0.78 | 0.78 |
|  | H3K4me3R1 | 0.60 | 0.69 | 1.00 | 0.96 | 0.76 | 0.70 |
|  | H3K4me3R2 | 0.64 | 0.72 | 0.96 | 1.00 | 0.74 | 0.69 |
|  | H3K9me2R1 | 0.66 | 0.77 | 0.76 | 0.74 | 1.00 | 0.93 |
|  | H3K9me2R2 | 0.67 | 0.78 | 0.70 | 0.69 | 0.93 | 1.00 |
